# Comparison of Electroacupuncture and Morphine-Mediated Analgesic Patterns in a Plantar Incision-Induced Pain Model

**DOI:** 10.1155/2014/659343

**Published:** 2014-11-02

**Authors:** Yen-Jing Zeng, Shih-Ying Tsai, Kuen-Bao Chen, Sheng-Feng Hsu, Julia Yi-Ru Chen, Yeong-Ray Wen

**Affiliations:** ^1^Graduate Institute of Clinical Medicine, College of Medicine, China Medical University, Taichung, Taiwan; ^2^Department of Anesthesiology, School of Medicine, China Medical University, Taichung, Taiwan; ^3^Department of Anesthesiology, China Medical University Hospital, No. 2, Yuh-Der Road, North District, Taichung 40447, Taiwan; ^4^Graduate Institute of Acupuncture Science, College of Chinese Medicine, China Medical University, Taichung, Taiwan; ^5^Department of Acupuncture, China Medical University Hospital, Taipei Branch, Taipei, Taiwan; ^6^Acupuncture Research Center, China Medical University, Taichung 40447, Taiwan; ^7^Department of Pediatrics, School of Medicine, Taipei Medical University, Taipei, Taiwan; ^8^Guang Li Biomedicine, Inc., Xizhi, New Taipei City, Taiwan; ^9^Pain Management and Research Center, China Medical University Hospital, Taichung 40447, Taiwan; ^10^Department of Anesthesiology, Shin-Kong Memorial Hospital, Taipei, Taiwan; ^11^Research Center for Chinese Medicine and Acupuncture, China Medical University, Taichung 40447, Taiwan

## Abstract

Electroacupuncture (EA) is a complementary therapy to improve morphine analgesia for postoperative pain, but underlying mechanism is not well-known. Herein, we investigated EA-induced analgesic effect in a plantar incision (PI) model in male Sprague-Dawley rats. PI was performed at the left hind paw. EA of 4 Hz and high intensity or sham needling was conducted at right ST36 prior to PI and repeated for another 2 days. Behavioral responses to mechanical and thermal stimuli, spinal phospho-ERK, and Fos expression were all analyzed. In additional groups, naloxone and morphine were administered to elucidate involvement of opioid receptors and for comparison with EA. EA pretreatment significantly reduced post-PI tactile allodynia for over 1 day; repeated treatments maintained analgesic effect. Intraperitoneal naloxone could reverse EA analgesia. Low-dose subcutaneous morphine (1 mg/kg) had stronger inhibitory effect on PI-induced allodynia than EA for 1 h. However, analgesic tolerance appeared after repeated morphine injections. Both EA and morphine could equally inhibit PI-induced p-ERK and Fos inductions. We conclude that though EA and morphine attenuate postincision pain through opioid receptor activations, daily EA treatments result in analgesic accumulation whereas daily morphine injections develop analgesic tolerance. Discrepant pathways and mechanisms underlying two analgesic means may account for the results.

## 1. Introduction

Surgery is a necessary evil which can be associated with complications such as unbearable pain. Poor postoperative pain control prolongs hospitalization days, increases perioperative morbidity, and causes chronic postoperative pain [1–4]. Different analgesic means have been introduced, and presently patient-controlled analgesia (PCA) with injectable morphine is the mainstay for postoperative pain. However, morphine-induced side effects and risks are always present, so multimodal analgesic strategies are therefore highly recommended for a purpose of opioid-sparing effect [5, 6]. Among choices, acupuncture or electroacupuncture (EA) has been suggested to be one of the available adjuvants to improve pain control quality [7–10].

Numerous studies demonstrated with convincible evidence that EA acts as a complementary treatment technique for various surgeries and invasive procedures (dental extraction, colonoscopy, and bronchoscopy) to reduce pain and related symptoms such as nausea, vomiting, and dizziness with or without PCA [8–13]. We had demonstrated benefits of pretreatment with EA before lower abdominal surgery in gynecological patients to reduce postoperative nausea and dizziness [11]. In basic researches, several lines of evidence indicate the antinociceptive effect of EA on inflammatory, neuropathic, and cancer pain models [14–18]. Strangely, there were so few animal studies investigating EA effect on postoperative pain up to today [19, 20].

To investigate mechanistic processes of postsurgical pain, Brennan et al. [21] had developed a rat model of incisional pain by creating a surgical incision in the plantar aspect of rat hind paw. This plantar incision (PI) leads to a battery of nociceptive responses, one of which is characterized by an increase of mechanical sensitivity that parallels the time course of postoperative pain in human [21, 22]. Using this model, different lines of studies concluded that incisional/surgical pain may be a unique entity which cannot be purely classified into neuropathic pain or inflammatory pain [23, 24]. For example, PI-induced central sensitization is more likely mediated by activation of non-*N*-methyl-D-aspartate (non-NMDA), metabotropic glutamate receptor (mGluR), and neurokinin-1 (NK-1) receptors in spinal dorsal horn [24–26], but not by* N*-methyl-D-aspartate (NMDA) receptors [24, 27] that play important roles in inflammatory hyperalgesia [28], and is essential to some subtypes of neuropathic pain [29]. On the other hand, nonsteroid inflammatory drugs (NSAIDs), morphine, and gabapentin are all effective in reducing paw incision-induced mechanical hyperalgesia and tactile allodynia, providing pharmacological evidence that both inflammatory and neuropathic mechanisms are involved in hyperalgesia/allodynia of this model. Because EA exerts analgesic effect via complex neurochemical and neuroanatomical mechanisms including endogenous opioid releases, spinal orexin 1 receptor-mediated nonopioid analgesia, serotonin/norepinephrine-mediated inhibitory controls, oxytocin-based central activity, and peripheral adenosine activations [30–38], it is worthy of investigating how EA affects PI-induced postoperative pain.

Both Fos expression and phosphorylation of extracellular signal-regulated kinase (ERK), a member of mitogen-activated protein kinase (MAPK), are induced in the spinal dorsal horns by peripheral nerve injury, inflammation, and paw incision and have been viewed as molecular evidence of neuronal excitation [39–42]. In most situations, p-ERK appears earlier than Fos protein, that is, minutes (p-ERK) versus hours (Fos), and is a better dynamic marker for central sensitization [41, 42]. Therefore, we examined behavioral and molecular changes responding to EA stimulation. The aim of this study is to explore the profiles of EA analgesia in animal incision-induced pain and to compare it with morphine injection in behavioral and molecular profiles. Part of our results had been reported as poster format in the annual meeting of World Institute of Pain in 2009.

## 2. Materials and Methods

### 2.1. Animal Preparation

Male Sprague-Dawley rats (250–300 gm; BioLASCO, Taipei, Taiwan) were used. Animals were housed in groups of two to three per cage at constant 22 ± 1°C, relative humidity of 40–60%, food and water* ad libitum*, and 12 h light/dark cycle environment for at least 5 days before the experiments to be acclimatized to the laboratory facility. All experiments were carried out under approval of the Institutional Animal Care and Utilization Committee, China Medical University, Taichung, Taiwan, and strictly followed the Guidelines for the Care and Use of Experimental Animals [43].

### 2.2. Electroacupuncture Stimulation

The electrical stimulation was modified by lab protocol [17, 33]. In brief, the rats were put into a transparent cylinder holder and were stably anaesthetized with 0.75% isoflurane in pure oxygen by a breathing circuit. EA was delivered through one pair of stainless steel needles (36 G, 0.22 mm in diameter) inserted at the right Zusanli acupoint (ST36) and another reference point 5 mm at right anterior tibial muscle along meridian. A constant current with square-wave pulses of 0.5 ms pulse width and 4 Hz was generated by a Grass S 88 stimulator and two Grass constant current units (Grass, West Warwick, RI, USA). The final stimulation intensity was escalated in a stepwise fashion to 10 times muscle twitch intensity, usually about 4-5 mA, for totally 30 min. Characteristic rhythmic dorsiflexion of the stimulated hind foot was always seen. In the sham-EA group, rats were inserted with needles without electrical stimulation. All rats recovered to a freely moving status within 5 min after anesthesia, indicating anesthetic effect was minimal. Our previous study also demonstrated that this procedure did not change baseline thresholds [17].

### 2.3. Plantar Incision (PI)

The plantar surgery had been reported before [21]. Under 2% isoflurane in oxygen, the plantar aspect of the left hind paw, contralateral to the EA side, was well-sterilized and placed through a hole in a sterile drape. A 1 cm longitudinal incision was made through skin and fascia at the paw, starting 0.5 cm from the proximal edge of the heel and extending toward the toes, and the plantaris muscle was longitudinally incised without cutting of origin and insertion. After hemostasis, the skin was apposed with 2 mattress sutures of 5-0 nylon. The incision was checked daily. Any rat with signs of wound infection was excluded from study. To avoid impact of antibiotics on pain response, all rats did not receive antibiotics injection.

### 2.4. Nociceptive Threshold Tests

To test tactile allodynia, a series of von Frey filaments with incremental stiffness (0.4, 1, 2.0, 4.0, 6.0, 10.0, 15.0, and 26.0 g) (Stoelting, Wood Dale, IL) was used. Animals were individually acclimated in chamber (10 × 10 × 20 cm) of plexiglas cage on an elevated iron mesh floor 20 min before testing. The filaments, starting from the 4.0 g filament, were perpendicularly applied from underneath the mesh openings to stimulate the plantar surface at medial aspect adjacent to the wound for 5-6 seconds for each filament. Stimulation was conducted in an up-down method [44] and results were transformed to a value of fifty percent withdrawal threshold [45]. Threshold value was an average of two test values at each time point. This protocol has been accepted in our previous papers [46, 47].

To test thermal hyperalgesia, animals were put in a plastic box placed on a glass plate prewarmed to constant 30°C (Plantar Test Apparatus, IITC, CA). The left plantar surface was exposed to a beam of radiant heat underneath the glass floor. The heat was adjusted to produce baseline latencies of 10–12 sec and a cut-off limit of 25 sec to prevent potential heat injury. Every withdrawal latency was an average of three tests, separated by a 5 min interval [48]. The experimenter who performed two behavioral tests was blind to the group allocation.

### 2.5. Immunohistochemistry and Quantification

Animals were overanesthetized with high-dose isoflurane and then transcardially perfused with saline at room temperature, followed by 4°C 4% paraformaldehyde in 0.1 M phosphate buffer (PB). The L4-5 spinal segments were carefully removed, postfixed overnight, and cryoprotected in 4°C 30% sucrose/PB for another 24–48 h. Before slicing, a hole was made at right spinal ventral horn by a fine needle as side marker. The tissues were cut by a cryostat and the free-floating sections (30 *μ*m) were stored in 0.1 M PB. After blocking with 2% normal goat serum containing 0.3% Triton X-100 at room temperature, all sections were incubated with either a rabbit anti-p-ERK MAPK primary antibody (1 : 400; Cell Signaling Technology, Beverly, MA) or polyclonal anti-Fos antibody (1 : 1500, Santa Cruz Biotechnology, CA) at 4°C for two nights. The sections were incubated with goat biotinylated anti-rabbit secondary antibody (1 : 200, Vector Laboratories, Burlingame, CA) for 2 h and subsequently reacted with avidin-biotin-peroxidase complex (Elite ABC kit, Vector Laboratories) for 1 h and reacted with diaminobenzidine-H_2_O_2_ solution (Peroxidase substrate kit, Vector Laboratory) with appropriate rinsing. Finally, sections were mounted onto gelatin-coated glass slides, air-dried, and coverslipped with Entellan mounting medium (Merck, Darmstadt, Germany).

Images of p-ERK-immunoreactive (p-ERK-ir) and Fos-immunoreactive (Fos-ir) cells were captured at a magnification of 20x under Nikon E600 (Tokyo, Japan) microscope in all cases. The immunoreactive cells in the dorsal laminae (I-V) were counted at randomly chosen sections and averaged from at least 6 sections for each lumbar segment. At least 4 rats in each group were included. The investigator who measured the staining was blind to group allocation.

### 2.6. Experimental Procedures

**Figure 1:**
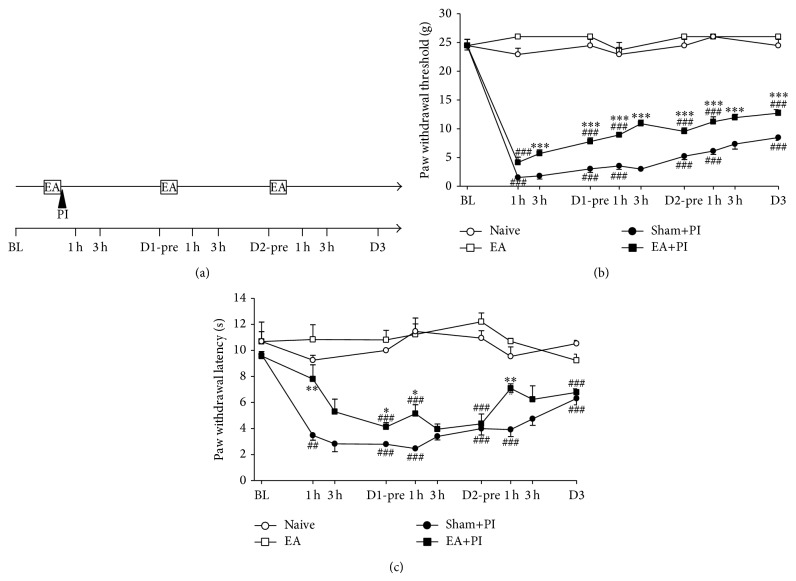
Effect of repeated daily EA treatments on PI-induced pain. (a) Summary of the protocols used in this experiment. BL: baseline on one day before PI; D: postplantar incision day; EA: electroacupuncture; h: hour; PI: plantar incision, marked by a solid triangle; pre: before daily EA treatment. (b) Mechanical allodynia, (c) heat hyperalgesia. Naïve: group without PI surgery or EA treatment, EA: group with repeated EA treatments, EA+PI: group for PI with repeated EA treatments, and Sham+PI: group for PI with repeated sham needle insertions; # < 0.05, ## < 0.01, ### < 0.001 groups versus Naïve; ∗ < 0.05, ∗∗ < 0.01, ∗∗∗ < 0.001 EA+PI versus Sham+PI; one-way ANOVA with Tukey's multiple comparison test; *N* = 4 (Naïve), 4 (EA), 7 (Sham+PI), and 9 (EA+PI).

**Figure 2:**
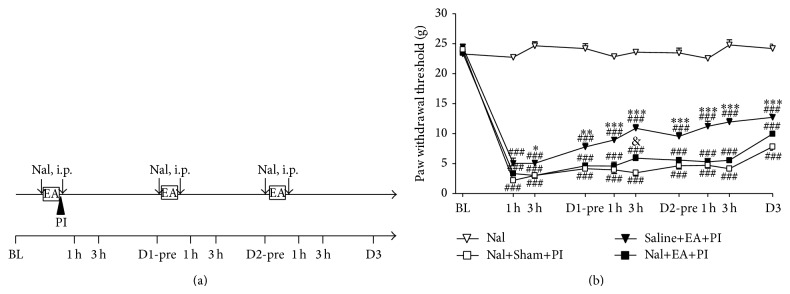
Intraperitoneal injections of naloxone (Nal) reversed EA analgesic effect on postincisional pain. (a) Summary of the protocols used in this experiment. Naloxone was injected at doses of 2 mg/kg immediately before anesthesia and 1 mg/kg immediately after PI and repeated the administration mode on D1 and D2. BL: baseline on one day before PI; D: postplantar incision day; EA: electroacupuncture; h: hour; i.p.: intraperitoneal injection; Nal: naloxone, marked with an arrow line; PI: plantar incision, marked with a solid triangle; pre: before daily Nal i.p. and EA treatment. (b) Intraperitoneal injections of naloxone almost completely antagonized EA analgesia to basal post-PI pain levels. Nal: group with i.p. naloxone, Saline+EA+PI: group for PI with repeated saline injections and EA treatments, Nal+Sham+PI: group for PI with repeated naloxone injections and sham needle insertion, and Nal+EA+PI: group for PI with repeated naloxone injections and EA treatments; ### < 0.001 groups versus Nal; & < 0.05 Nal+EA+PI versus Nal+Sham+PI; ∗ < 0.05, ∗∗ < 0.01, ∗∗∗ < 0.001 Saline+EA+PI versus Nal+EA+PI; one-way ANOVA with Tukey's multiple comparison test; *N* = 4 (Nal), 7 (Nal+Sham+PI), 7 (Nal+EA+PI), and 6 (Saline+EA+PI).

**Figure 3:**
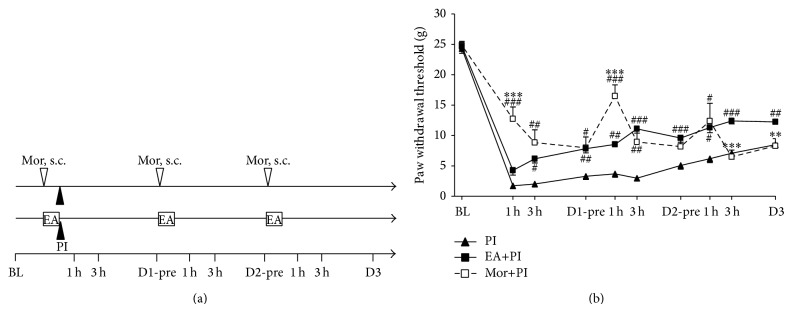
Comparison of analgesic patterns between EA and subcutaneous injection of morphine in incisional pain. (a) Summary of the protocols used in this experiment. BL: baseline on one day before PI; D: postplantar incision day; EA: electroacupuncture; h: hour; s.c.: subcutaneous injection; Mor: morphine, marked with a blank triangle; PI: plantar incision, marked with a solid triangle; pre: before daily Mor i.p. and EA treatment. (b) Mechanical allodynia, PI: group for PI surgery, EA+PI: group for PI with repeated EA treatments, and Mor+PI: group for PI with repeated morphine s.c. injections; # < 0.05, ## < 0.01, ### < 0.001 groups versus PI; ∗∗ < 0.01, ∗∗∗ < 0.001 Mor+PI versus EA+PI; one-way ANOVA with Tukey's multiple comparison test. *N* = 6 (PI), 9 (EA+PI), and 6 (Mor+PI).

The study designs are plotted in Figures 1–3 subgraph (a) individually. For EA study, rats were allocated into the Naïve, EA, Sham+PI, or EA+PI group. EA or sham treatment was conducted before PI, post-PI Day 1 (D1) and D2. Behavioral tests were performed before PI, at 1, 3 h after daily EA, and post-PI D3.

Second, opioid receptor-mediated effect was investigated by intraperitoneal (i.p.) naloxone (Nal) injection. Naloxone was injected at doses of 2 mg/kg immediately before anesthesia and 1 mg/kg immediately after PI and repeated the administration mode on D1 and D2. The dose is based on previous studies [17, 33]. Rats were divided into four groups: the Nal, Saline+EA+PI, Nal+Sham+PI, or Nal+EA+PI group.

Last, we compared analgesic patterns between EA and morphine injection. Rats in the Mor+PI group received subcutaneous (s.c.) injection of 1 mg/kg morphine (Mor) and sham needling at 30 min before PI and at post-PI D1 and D2. The EA+PI group received EA stimulation and s.c. saline injection of the identical volume. We injected morphine at subcutaneous tissue over right thigh because the rat was placed in a restrained tube for anesthesia and i.p. technique became difficult and unsecure.

Some rats in the EA treatment study, at least 4 in a group, were sacrificed for immunohistochemical analysis of phosphorylated ERK (p-ERK) and Fos. The inductions of p-ERK and Fos were analyzed in samples from L4-5 spinal dorsal horn at 30 min and 3 h, respectively, after PI.

All study protocols were standardized, and baselines of mechanical and thermal thresholds were measured from at least 2 days before surgery to eliminate the hyper- or hyposensitive rats. There were at least 6 rats at each group for behavioral tests. Because our anesthetic apparatus can anesthetize maximally three rats at a time, we always included one sham and one EA rat at each test to minimize background biases.

### 2.7. Statistical Analysis

All the results were expressed as mean ± SEM (standard error of the mean). Two-way analysis of variance (ANOVA) was conducted to analyze the influence of factors of time and treatments. Data from behavioral tests and the mean numbers of immunoreactive cells were analyzed by one-way ANOVA with post hoc Tukey's test (PASW Statistics for Windows, Version 18.0. Chicago: SPSS Inc). *P* < 0.05 was considered statistically significant.

## 3. Results

### 3.1. Repeated EA Treatments Attenuate PI-Induced Nociceptive Responses

In consistence with previous studies and our studies [21, 49], plantar incision evidently decreased tactile and thermal withdrawal thresholds (Figures 1(b) and 1(c)). The allodynic thresholds to von Fey stimulation in the Sham+PI group were reduced from preoperative 24.40 ± 0.69 g to 1.53 ± 0.39 g 1 h after surgery and persisted low till post-PI D3. The EA stimulation under anesthesia did not alter basal thresholds as comparing the EA group with the Naïve group, suggesting that EA did not affect peripheral nociceptive sensitivity in a normal condition. Because we found post-PI pain returned to baseline on the 5th post-op day [17], we did not measure pain responses after D3 in this study.

EA stimulation significantly prevented and attenuated postoperative aversive responses. After EA pretreatment, the EA+PI group showed higher mechanical thresholds than the Sham+PI group at the post-PI 1 h (EA+PI versus Sham+PI: 4.13 ± 0.93 g versus 1.53 ± 0.39 g, *P* = 0.059), 3 h (5.69 ± 0.66 g versus 1.80 ± 0.47 g *P* < 0.001), and 1 day after (7.77 ± 0.66 g versus 3.03 ± 0.52 g, *P* < 0.001). Daily EA stimulations sustained analgesic effect. Significant differences in tactile allodynia were shown at all post-PI time points afterwards (Figure 1(b)). In Figure 1(c), EA shortly prevented thermal hyperalgesia at post-PI 1 h (7.80 ± 1.10 g versus 3.47 ± 0.37 g, *P* < 0.01), but no effects after 3 h, and had only 1 h effect after each EA stimulation. These data suggest that EA did not produce a prolonged effect on heat hyperalgesia by repeated treatments, whereas repeated EA seemed to produce an accumulating effect on mechanical analgesia (Figures 1(b) and 1(c)).

### 3.2. EA Analgesia Is Opioid-Dependent but Differs from Morphine-Induced Analgesic Pattern

Injection of high-dose naloxone (total 3 mg/kg, i.p.) did not alter normal withdrawal thresholds in the naive rats (the Nal group, Figure 2(b)). Repeated i.p. naloxone injections before and after daily EA completely reversed EA analgesia (the Nal+EA+PI versus the Saline+EA+PI, *P* < 0.05 for almost all time points, Figure 2(b)). Naloxone significantly antagonized EA-induced analgesic effect, which suggests that an opioid-dependent analgesic mechanism is involved.

Based on the above findings, we further compared effects of EA and morphine in PI model (Figures 3(a) and 3(b)). Morphine at a dose of 1 mg/kg produced stronger inhibition on PI-induced allodynia than EA at post-PI 1 h (Mor+PI versus EA+PI: 12.73 ± 1.97 g versus 4.26 ± 0.77 g, *P* < 0.001) and 3 h (8.83 ± 2.10 g versus 6.17 ± 0.69 g, *P* = 0.212). The morphine dose is based on our study that EA at 10× basal muscle-twitch intensity is equipotent to morphine 1 mg/kg [17]. We also found the following: first, morphine has stronger effect at 1 h, whereas EA effect escalates over time; second, morphine effect is stronger than EA before Day 2 but is lower than EA on Day 3; and third, the Mor+PI group actually suffered the same pain intensity as the PI group after repeated morphine injections (Figure 3(b)). In summary, repeated morphine injections develop analgesic tolerance, whereas the intense EA keeps increasing inhibition on PI-induced mechanical hypersensitivity.

### 3.3. EA and Morphine Inhibit Post-PI ERK Activation and Fos Expression in the Spinal Dorsal Horn

**Figure 4:**
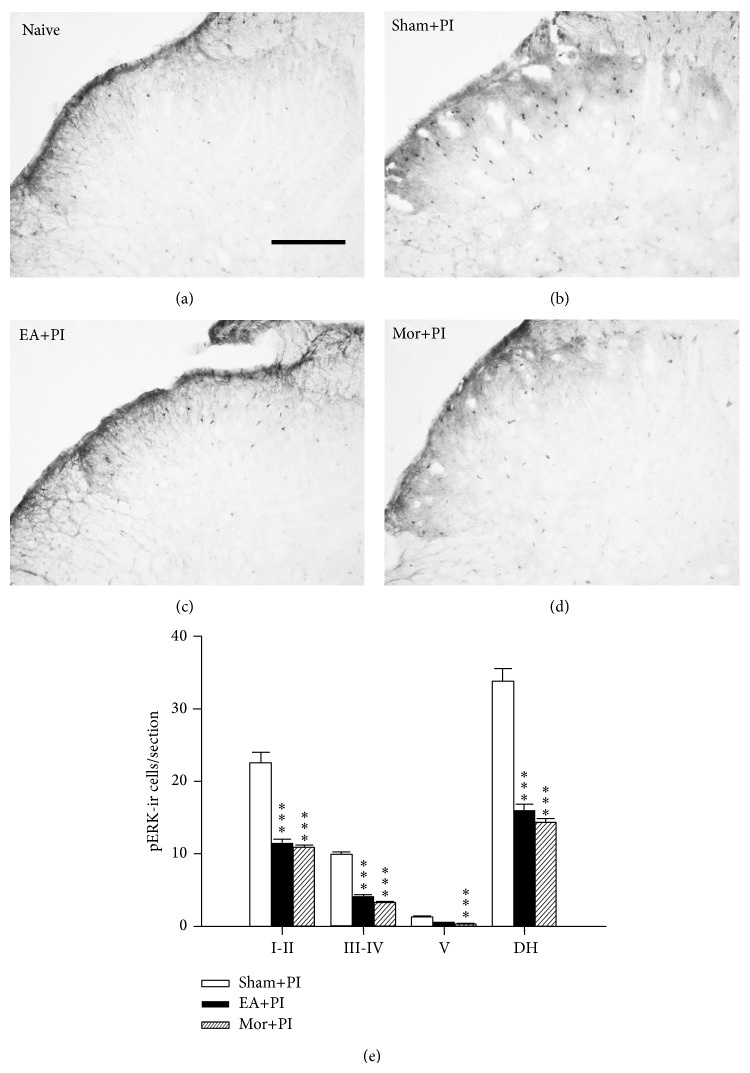
Effect of EA and morphine on ERK activation 30 minutes after PI in the lumbar (L4 or L5) spinal dorsal horns. (a)–(d) are section slices showing spinal dorsal horn at the PI-ipsilateral side in the Naïve group ((a) rats without any operation or treatment), Sham+PI group ((b) rats with PI and sham needling and saline injection), EA+PI group ((c) rats with PI and pretreated EA), and Mor+PI group ((d) rats with PI and preinjected morphine). (e) Numbers of pERK-immunoreactive cells by lamina in the spinal dorsal horn among groups. Note no differences between the EA+PI and Mor+PI groups. Laminas I-II: superficial dorsal horn, laminas III-IV: middle dorsal horn, and lamina V: deep dorsal horn. DH: dorsal horn; ∗∗∗ < 0.001 groups versus Sham+PI, one-way ANOVA with Tukey's multiple comparison test; *N* = 4 for each group, respectively. Scale bar = 100 *μ*m.

**Figure 5:**
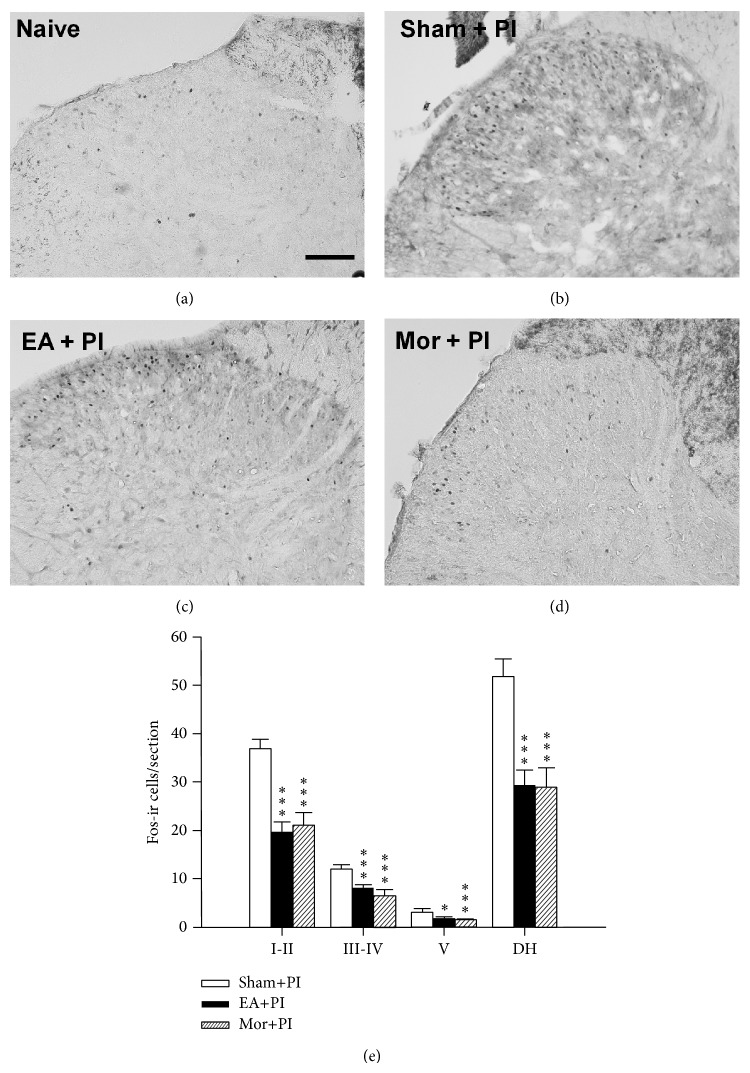
Effect of EA and morphine on Fos expression 3 hr after PI in the lumbar (L4 or L5) spinal dorsal horns. (a)–(d) are section slices showing spinal dorsal horn at the PI-ipsilateral side in the Naïve group ((a) rats without any operation or treatment), Sham+PI group ((b) rats with PI and sham needling and saline injection), EA+PI group ((c) rats with PI and pretreated EA), and Mor+PI group ((d) rats with PI and preinjected morphine). (e) Numbers of Fos-immunoreactive cells by lamina in the spinal dorsal horn among groups. Note no differences between the EA+PI and Mor+PI groups. Laminas I-II: superficial dorsal horn, laminas III-IV: middle dorsal horn, and lamina V: deep dorsal horn. DH: dorsal horn; ∗ < 0.05, ∗∗∗ < 0.001 groups versus Sham+PI, one-way ANOVA with Tukey's multiple comparison test; *N* = 4 for each group. Scale bar = 100 *μ*m.

The naive rats without PI or EA presented very few ERK activation or Fos expression in the spinal dorsal horn (Figures 4(a) and 5(a)); however, strong expression of p-ERK-ir and Fos-ir cells was observed postoperatively (Figures 4(b) and 5(b)). In Figure 4, p-ERK-ir cells were evidently shown at 30 min after PI and were predominantly found in the laminae I-II (Figures 4(b) and 4(e)) compared to the naive group (Figures 4(a) and 4(e)). Different from Fos expression, ERK activation after PI was similarly seen at bilateral dorsal horns (figure not shown). Both EA and morphine treatments reduced p-ERK-ir cell amounts in spinal dorsal horn in comparison with that in the Sham+PI group (Figures 4(b)–4(d)). Notably, p-ERK were significantly depressed in the superficial (laminae I-II) and middle (lamina III-IV) dorsal horns (Figure 4(e)). Meanwhile, there is no significant difference in numbers of immunoreactive cells between the EA+PI group and the Mor+PI group (Figure 4(e)), showing discrepant results from the behavioral observation (Figure 3(b)).

In Figure 5, most of the Fos protein appeared in the superficial dorsal horns (laminae I-II) of ipsilateral side 3 h after PI and were intensely clustered at the outer one-half lamina (Figure 5(b)). EA and morphine treatments markedly depressed the Fos expression in the dorsal horn (Figures 5(c)–5(e)). By calculation, EA and morphine significantly reduced Fos-ir cells in the dorsal horns to levels of over 40% reduction (EA+PI: 26.39 ± 3.09 and Mor+PI: 28.95 ± 3.93 versus Sham+PI: 51.84 ± 3.63, *P* < 0.001 individually, Figure 5(e)). The strongest reductions after both treatments are present at the superficial laminae. Similar to p-ERK finding, there is no significant difference in numbers of immunoreactive cells between the EA+PI group and the Mor+PI groups (Figure 5(e)).

## 4. Discussion

We present in the study that EA stimulation is efficacious in reducing incision-induced tactile allodynia and heat hyperalgesia, as well as suppressing nociception-activated ERK phosphorylation and Fos expression in the spinal cord. Daily EA can maintain the analgesic effect. Most importantly, repeated EA does not show analgesic tolerance, as observed in the morphine treatment group.

### 4.1. Compare Our EA Results with the Previous EA Studies in a PI Model

The first study of EA effect in rat PI model was done by Oliveira and Prado [19] who reported EA reduced incision-induced mechanical hypersensitivity. However, we provided more information in this study. First, we used different EA parameters from Oliveira's study. Comparing the details, they used 4 Hz EA of low intensity (the lowest intensity that produced hind limb muscle contraction) and stimulated acupoint at the same side of PI, whereas we gave 4 Hz EA of high intensity (10× muscle twitch) at the contralateral side. Our preliminary data using low intensity EA at the other side of PI injury show no antiallodynic effect. It is possible that EA at the injured side as Oliveira's study could exert stronger analgesia by combining peripheral and central EA effects [37]. Second, we demonstrated EA had short-term effect on thermal hyperalgesia in PI model, which was not investigated in previous study. Third, because EA effect is completely antagonized by systemic naloxone, it proved that peripheral and central (including spinal and brain) opioid receptor-mediated analgesic action is involved. Our undisclosed data showed intrathecal morphine, at a dose of 20 *μ*g, could also completely reverse EA effect and suggests a strong spinal mechanism. However, the supraspinal opioid actions cannot only be excluded but should be critically considered in this model for reasons in the following paragraphs.

The EA intensity we used is relatively strong but not intolerable. It is higher than those in most awake animal EA studies [16, 50] and clinical human treatment (usually below 3 mA) but is still lower than those in other studies (about 10–20 mA) [17, 51]. We found this strong EA neither altered the withdrawal thresholds by von Frey fibers or thermal test in normal rats nor induced higher Fos expression in the stimulated dorsal horns [17]. However, we believed that this strong EA stimulation can be safely and effectively applied to anesthetized patients for ameliorating postsurgical pain [11, 52].

### 4.2. EA Effect Is Mediated through Opioid Releases via Ascending-Descending Circuits

High dose of i.p. naloxone antagonizes EA-produced long-lasting analgesia. EA effect in this study cannot be a purely homosegmental inhibition (such as Gate control) in that EA stimulated at the opposite side of paw incision. It is likely that intense EA at the right hind limb sends information ascendingly to trigger the supraspinal structures (e.g., brainstem, midbrain, and cortex [53]) and activates descending inhibitory pathways to diffusely downregulate spinal nociceptive processes. These descending pathways, which need neurotransmitters such as serotonin [35, 54], norepinephrine [35, 36], and/or oxytocin [34], may terminate at opioid-containing interneurons in the superficial laminae to release opioid peptides from these cells [55–57]. Distribution of opioid receptors in the spinal synapses plays important roles because PI induces strong p-ERK and Fos at the spinal cord levels which controls spinal sensitization and subsequent long-term neuroplasticity. Unfortunately, it is unclear in this study if EA stimulation at the ipsilateral side could yield additional analgesic effect by local activation of segmental circuit.

The reduction of Fos and p-ERK expression in the spinal dorsal horn supports spinal inhibitory mechanism exerted by EA and morphine treatments. We examined two markers at two respective time points because spinal ERK phosphorylation occurs early after peripheral injury (within tens of min) and Fos protein is an end product of immediately early gene c-*fos* expression for hours [41]. We found that both markers were reduced to a similar level, suggesting that these two means produced equivalent inhibitory strength. Our results also suggest that either EA or morphine has ability to suppress central sensitization mediated by ERK activations early after PI. However, inhibition of signal molecules does not seem to correlate with behavioral findings because morphine led to stronger analgesia than EA during the same period. While c-Fos is often induced in the nuclei of neurons, p-ERK can be induced in different subcellular structures of neurons and even in spinal cord microglia and astrocytes [41, 58]. Spinal glia-mediated neuroinflammation can enhance nociceptive sensitization early after PI and may ultimately differentiate the analgesic response to repeated EA from repeated morphine treatments in that chronic morphine may activate, rather than inhibit, glial activations [59, 60].

### 4.3. EA Analgesic Mechanisms Are Not the Same as Morphine Treatment

Based on these deductions, it is rational that EA-triggered opioid action can gradually increase and persist by activating complex spinal and supraspinal mechanisms and accumulating adequate endogenous opioids, compared to the immediate and short effect of injected morphine which passes absorption, circulation to reach spinal cord and brain targets, and metabolism. We injected 1 mg/kg morphine because we had demonstrated that morphine at 1 mg/kg is equipotent to EA of 10× basal intensity [17]. Though both analgesic means are opioid receptor-dependent, EA is somewhat different because secretions of neuropeptides like endorphin, enkephalin, or endomorphin [30–32] after EA stimulation require a transcription-translational process. In addition, repeated EA may activate mechanisms other than opioid system and positively enhance EA analgesic maintenance [32, 35].

In this study, the first morphine injection produced strong and lasting antiallodynic potency, the second injection exerted strong but short effect, and then the third injection had only low and short effect. It is undoubted that the morphine group developed analgesic tolerance to injections of the same morphine doses, but not analgesic accumulation as the EA group did. Though EA tolerance could happen after chronic daily EA stimulations [61–63], most of those studies used high frequency (100 Hz) instead of low frequency EA in this study. However, we actually observed escalating EA analgesic effect in this study. Possible explanations include that repeated low-frequent EA is less prone to develop acute tolerance than morphine, interactions between opioid and nonopioid pathways, and spinal microglial inhibition [16, 59, 61]. Nevertheless, this advantage over morphine merits more clinical considerations of EA in chronic pain therapies [64].

Growing evidence supports EA efficacy is stronger in pathological conditions than in a normal control. We found EA produced prolonged analgesia, much longer than duration of tail flick inhibition in the naïve rats (about 90–120 min) using the same EA setting [17]. In rats with complete Freud's adjuvant-induced inflammation, nociceptive neurons in the ipsilateral dorsal horn have expansion of receptive field size and exhibit hyperexcitability and hyperresponsiveness [50]. Further, persistent inflammation upregulates opioid receptors in the central terminals of primary nociceptive afferents and postsynaptic projecting neurons and alters sensitivities of opioid receptor subtypes to EA [65]. Therefore, EA exerts a long antihyperalgesic effect via *μ*- and *δ*-receptor activation, but not *κ*-mediated function in pain model [66, 67]. Taken together, it is deductive that EA can be additive to morphine treatment through different mechanisms to achieve an adequate pain control quality.

## 5. Conclusions

In conclusion, intense EA stimulation suppressed incision-induced pain in a rat surgical pain model via an opioid-dependent analgesic effect. Particularly, repeated EA did not show analgesic tolerance as daily morphine administrations. Inhibition of Fos expression and ERK activity in the spinal dorsal horn implicates an important role of spinal mechanisms in EA analgesia. This preclinical study opens an alternative view on EA mechanisms.
